# Synergistic Effects of Thiosemicarbazides with Clinical Drugs against *S. aureus*

**DOI:** 10.3390/molecules25102302

**Published:** 2020-05-14

**Authors:** Beata Chudzik-Rząd, Anna Malm, Nazar Trotsko, Monika Wujec, Tomasz Plech, Agata Paneth

**Affiliations:** 1 Department of Pharmaceutical Microbiology with Laboratory for Microbiological Diagnostics, Faculty of Pharmacy, Medical University of Lublin, Chodźki 1, 20-093 Lublin, Poland; BeChudz@poczta.fm (B.C.-R.); anna.malm@umlub.pl (A.M.); 2 Department of Organic Chemistry, Faculty of Pharmacy, Medical University of Lublin, Chodzki 4a, 20-093 Lublin, Poland; nazar.trotsko@umlub.pl (N.T.); monika.wujec@umlub.pl (M.W.); 3 Department of Pharmacology, Faculty of Health Sciences, Medical University of Lublin, Chodźki 4a, 20-093 Lublin, Poland; tomasz.plech@umlub.pl

**Keywords:** thiosemicarbazides, antibacterial activity, synergistic effect, bacterial topoisomerases

## Abstract

Antimicrobial resistance spurred by the overuse and misuse of antibiotics is a major global health concern, and of the Gram positive bacteria, *S. aureus* is a leading cause of mortality and morbidity. Alternative strategies to treat *S. aureus* infections, such as combination therapy, are urgently needed. In this study, a checkerboard method was used to evaluate synergistic interactions between nine thiosemicarbazides (4-benzoyl-1-(2,3-dichloro-benzoyl)thiosemicarbazides **1**–**5** and 4-aryl-1-(2-fluorobenzoyl)thiosemicarbazides **6**–**9**) and conventional antibiotics against *S. aureus* ATCC 25923, which were determined as the fractional inhibitory concentration indices (FICIs). For these experiments, amoxicillin, gentamicin, levofloxacin, linezolid, and vancomycin were selected to represent the five antimicrobial classes most commonly used in clinical practice. With one exception of **7**-vancomycin combination, none of the forty-five thiosemicarbazide-antibiotic combinations tested had an antagonistic effect, showing promising results with respect to a combination therapy. The synergic effect was observed for the **2**-linezolid, **4**-levofloxacin, **5**-linezolid, **6**-gentamicin, **6**-linezolid, and **7**-levofloxacin combinations. No interactions were seen in combination of the thiosemicarbazide with gentamicin or vancomycin, whereas all combinations with linezolid acted in additive or synergism, except for **6**-gentamicin and **7**-linezolid. The 4-(4-chlorophenyl)-1-(2-fluorobenzoyl)thiosemicarbazide **6** showed a clear preference for the potency; it affected synergistically in combinations with gentamicin or linezolid and additively in combinations with amoxicillin, levofloxacin, or vancomycin. In further studies, the inhibitory potency of the thiosemicarbazides against *S. aureus* DNA gyrase and topoisomerase IV was examined to clarify the molecular mechanism involved in their synergistic effect in combination with levofloxacin. The most potent synergist **6** at concentration of 100 µM was able to inhibit ~50% activity of *S. aureus* DNA gyrase, thereby suggesting that its anti-gyrase activity, although weak, may be a possible factor contributing to its synergism effect in combination with linezolid or gentamycin.

## 1. Introduction

Antimicrobial resistance spurred by the overuse and misuse of antibiotics is a major global health concern, and of the Gram positive bacteria, *S. aureus* is a leading cause of mortality and morbidity [1,2,3,4,5,6,7]. The important clinical *S. aureus* infections are bacteraemia, infective endocarditis, as well as skin and soft tissue, osteoarticular, pleuropulmonary, and device-related infections. Other clinical manifestations of *S. aureus* infections include epidural abscess, meningitis, toxic shock syndrome, and urinary tract infections [8,9]. Originally, *S. aureus* was predominately a nosocomial pathogen. Over time, epidemiologically distinct clones emerged in the community setting to cause a global public health concern. In fact, *S. aureus* deploys a specific combinations of virulence factors, such as adhesins, toxins, and immunomodulatory molecules, that facilitate infection of host tissues and evading from host immune response [10,11]. The ability to resist the activity of multiple antibiotics classes makes *S. aureus* difficult to treat. Indeed, over decades, *S. aureus* isolates have developed resistance to several classes of antibiotics, like *β*-lactams, fluoroquinolones, macrolides, glycopeptides, and oxazolidinones [12,13]. Especially, emergence and spread of methicillin-resistant *S. aureus* (MRSA) resulted in high morbidity and mortality which demands prompted clinical attention [14,15]. Vancomycin remained as one of the last resort therapies to tackle these strains for years, but its slow bactericidal activity, low tissue penetration, and the emergence of resistance restricted its clinical utility [16,17,18,19]. Although daptomycin remains as one of the main treatment options for MRSA, sporadic resistance cases reported in patients treated with daptomycin are a growing concern [20,21]. This situation has resulted in an urgent need to identify and develop novel antibacterial candidates to treat infections caused by *S. aureus*.

To explore and discover novel chemical scaffold with potent antibacterial activity, a library of about 1000 thiosemicarbazides constructed in our lab was tested in the broth microdilution assay against the panel of reference Gram positive and Gram negative bacteria. The most potent of them with 4-benzoylthiosemicarbazide scaffold inhibited the growth of *S. aureus* clinical isolates at concentrations equipotent or even lower compared to ampicillin, vancomycin, streptomycin, and nitrofurantoin [22]. In addition, they gave successful inhibition effect against growth and biofilm formation by clinical isolates of MSSA (methicillin-sensitive *S. aureus*), MRSA, and MDR-MRSA (multidrug-resistant MRSA) [23]. Thus, the unique chemical scaffold, low cytotoxicity and effectiveness of thiosemicarbazides against *S. aureus* spurred us to further explore their antibacterial potential and safety profile. Several studies published in recent years have shown that the combination therapy is one of the important strategies to improve efficacy and bioavailability, as well as treat mixed diseases in the clinic. One example is the study by Saisubramanian and co-workers [24] describing the potentiated effect of ciprofloxacin in combination with benzochromene derivatives against drug resistant strains of *S. aureus*. The rationale for the combination therapy includes synergistic effects in vitro, overcome multi-drug resistance, prevention of dissemination by more rapid bactericidal activity, optimized biofilm activity, and intracellular penetration. Taking this into account, we performed the microdilution broth checkerboard assay to evaluate synergism effects between five 4-benzoylthiosemicarbazides with previously confirmed antibacterial potential [23] and conventional antibiotics against *S. aureus* ATCC 25923. For these experiments four 4-arylthiosemicarbazides were also included for comparison, whereas amoxicillin, gentamicin, levofloxacin, linezolid, and vancomycin were selected to represent the five antimicrobial classes most commonly used in clinical practice. Our results indicate that is possible to establish a combination therapy between these substances, since none of the combinations tested had an antagonistic effect, except for one. Although most combinations had indifferent or additive effects, six of them showed synergistic interactions. Since combinations of antibacterial agents with similar mechanisms of action or those that influence the same target exert synergistic effects more readily than others, in further studies, the inhibitory potency of the thiosemicarbazides against bacterial topoisomerases was examined to clarify the molecular mechanism involved in their synergistic effect in combination with levofloxacin. The results from these experiments are presented in this paper.

## 2. Results and Discussion

### 2.1. Rationale and Synthesis

**Scheme 1:**
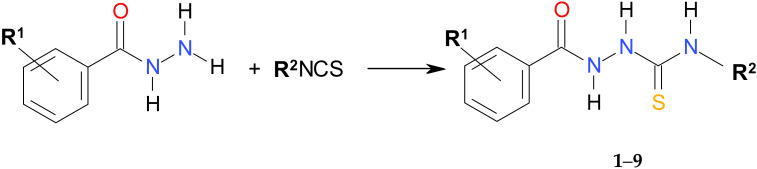
Synthetic route for 4-benzoylthiosemicarbazides (**1**–**5**) and 4-arylthiosemicarbazides (**6**–**9**). **R^1^** = 2,3-diCl (**1**–**5**), 2-F (**6**–**9**); **R^2^** = 2-MeC(=O)Ph (**1**), 3-MeC(=O)Ph (**2**), 2-ClC(=O)Ph (**3**), 4-ClC(=O)Ph (**4**), 4-NO_2_C(=O)Ph (**5**), 4-ClPh (**6**), 3-ClPh (**7**), 3-CF_3_Ph (**8**), and 3-BrPh (**9**). For full structures of **1**–**9**, see Table 2.

So far, the most promising antibacterial agents that emerged from our studies on antibacterial potency of thiosemicarbazides [20,21,23,25,26] are those with 4-benzoyl-1-(2,3-dichlorobenzoyl) thiosemicarbazide scaffold. The most potent of them, **1**–**5**, have been shown to possess potent, broad spectrum of antibacterial activities against Gram positive strains with *minimal inhibitory concentrations* (*MICs*) between 0.49 and 15.63 µg/mL, thereby indicating, in most cases, equipotent or even more effective action than cefuroxime [23]. In further studies, the inhibitory efficacy of *para*-nitro compound **5** on planktonic cells and biofilms formation in clinical isolates of *S. aureus* was also tested, and it was found that the compound was able to eradicate MSSA and MRSA biofilms at low concentrations, with minimal biofilm inhibitory concentrations (MBICs) between 7.82 and 15.63 µg/mL [23]. Thus, to further explore antibacterial potential of potent thiosemicarbazides **1**–**5**, their synergistic effects in combination with clinical drugs were tested, and the results of these studies are presented in this paper. For these studies, four 4-arylthiosemicarbazides **6**–**9** with much weaker antibacterial potency against reference strains of Gram-positive bacteria as compared to **1**–**5** were also included for comparison (Table 1). All compounds **1**–**9** were prepared using an established route described elsewhere [22,23], in one-step reaction of appropriate hydrazide with aryl or benzoyl isothiocyanate (Scheme 1). 

### 2.2. Cytotoxicity Assay 

A critical factor in research and development of new antibacterial agents is their abilities to selectively target bacterial cells without toxicity to mammalian cells. Thus, in order to test if antibacterial activity of title thiosemicarbazides is not due to their general toxicity, their effect on mammalian cell viability was assessed using human foreskin fibroblast Hs27 cells (CRL-1634) via MTT assay. In Table 2, the data are presented as the relative viability at the concentration of 25 μg/mL compared to control vehicle-treated samples (100% viability) because except of **2**, the compounds reached their solubility limit before their IC_50_ values could be determined. The results confirmed that all tested thiosemicarbazides produced very limited effects on the cellular viabilities of Hs27 cells at the concentration of 25 μg/mL after 24 h of treatment. Important to note, all potent antibacterial agents with 4-benzoylthiosemicarbazide core structure (**1**–**5**) reached their effective inhibitory effect against *S. aureus* ATCC 25923 strain much before the concentration at which their cytotoxic affect against Hs27 cell line could be observed. The second important observation from the cytotoxic assay is that the trend in antibacterial potency against *S. aureus* ATCC 25923; **5** (MIC 1.95 µg/mL) > **2** (MIC 7.81 µg/mL) > **1** = **3** = **8** (MIC 15.63 µg/mL) > **4** (MIC 31.25 µg/mL) > **7** = **9** (MIC 62.5 µg/mL) > **6** (MIC 500 µg/mL) is fortunately not linked directly with cytotoxic effect. Indeed, the weakest antibacterial agents **6**, **7**, and **9** differ substantially in their cytotoxicity; the compound **9** was the most cytotoxic, whereas **6** and **7** were at the same least cytotoxic against Hs27 cells among all thiosemicarbazides tested. 

For the compound with the best solubility (**2**), its cytotoxicity against L929 and HeLa cells was presented previously [23]. The cytotoxicity was expressed as rigorous IC_30_ values, defined as the highest dilutions of test samples that cause 30% or greater destruction of cells. According to these results, the compound **2** gave the IC_30_ value of 30.78 µg/mL against the L929 cell line and the IC_30_ value of 30.97 µg/mL against the HeLa cell line. Thus, as evidenced by these data, the compound **2** display antibacterial effect against *S. aureus* ATCC 25923 at non-toxic concentrations in mammalian cells, with the estimated selectivity indexes SI (SI = IC_30_/MIC) against L929 and HeLa cell lines 3.94 and 3.97, respectively.

### 2.3. Synergy Assay

The microdilution checkerboard method was used to evaluate the synergistic effects between the thiosemicarbazides **1**–**9** and antibiotics, which were determined as the fractional inhibitory concentration indices (FICIs). For these experiments, amoxicillin, gentamicin, levofloxacin, linezolid, and vancomycin were selected to represent the five antimicrobial classes most commonly used in clinical practice. In this way, a total of 45 combinations of thiosemicarbazide-amoxicillin, thiosemicarbazide-gentamicin, thiosemicarbazide-levofloxacin, thiosemicarbazide-linezolid, and thiosemicarbazide-vancomycin against *S. aureus* ATCC 25923 were tested to identify in vitro synergy effects.

As presented in Table 3, when the 4-benzoylthiosemicarbazide **1** with electron-donating *ortho*-methyl substitution was combined with tested antibiotics, an additive effect with amoxicillin or linezolid was observed, whereas the other three **1**-antibiotic combinations showed no interactions. Moving the *ortho*-methyl group of **1** to the *meta*-position to give **2** results in synergistic effect of the **2**-linezolid combination and additive effect of the **2**-amoxicillin combination. As for compound **1**, the other three **2**-antibiotic combinations showed no interactions. In turn, replacing the electron-donating *ortho*-methyl group of **1** with the electron-withdrawing *ortho*-chloro group results in the compound **3** that affected additively by **3**-amoxicillin, **3**-gentamicin, **3**-levofloxacin, and **3**-linezolid combinations, while no interaction were seen when **3** and vancomycin was combined. Moving the *ortho*-chloro group of **3** to the *para*-position to give **4** results in synergistic effect of the **4**-levofloxacin combination and additive effect of **4**-amoxicillin and **4**-linezolid combinations. As for other compounds tested, the combination of **4** with vancomycin did not display any synergism nor additivity. Finally, replacing the *para*-chloro group in **4** with the electron-withdrawing *para*-nitro results in analogue **5** that affected synergistically by **5**-linezolid combination, whereas the other four **5**-antibiotic combinations showed no interactions.

Further investigations were performed for set of 4-arylthiosemicarbazides **6**–**9**. As shown in Table 3, the *para*-chloro compound **6** with synergistic interactions in combinations with gentamicin or linezolid and additive interactions in combinations with amoxicillin, levofloxacin, or vancomycin showed a clear preference for the potency among this series. Its *meta* isomer **7** affected synergistically in combination with levofloxacin and additively in combination with amoxicillin, whereas no interactions or even antagonistic were detected for its combinations with gentamicin, linezolid, or vancomycin, respectively. In turn, the other two 4-arylthiosemicarbazides with *meta* electron-withdrawing trifluoromethyl **8** or bromo **9** substitutions showed the same trend in potency; they effected additively with levofloxacin or linezolid and indifferently in the combinations with amoxicillin, gentamicin, or vancomycin. 

Overall, except for **7**-vancomycin combination, none of the forty-five thiosemicarbazide-antibiotic combinations tested had an antagonistic effect, showing promising results with respect to a combination therapy. Among them, the synergic effect was observed for the **2**-linezolid, **4**-levofloxacin, **5**-linezolid, **6**-gentamicin, **6**-linezolid, and **7**-levofloxacin combinations. Except for **3**, **6**, and **7**, no interactions were seen when gentamicin or vancomycin with **1**–**9** were combined, whereas all thiosemicarbazide-linezolid combinations acted in additive or synergism, except of **7**-linezolid combination. The 4-arylthiosemicarbazide with *para*-chloro substitution **6** showed a clear preference for the potency among all the thiosemicarbazides tested; it affected synergistically in combinations with gentamicin or linezolid and additively in combinations with amoxicillin, levofloxacin, or vancomycin. It is important to note, however, although the checkerboard broth dilution method is suitable for in vitro experiments aiming to mimic in vivo conditions [27], in vitro FIC index is still unlikely to predict in vivo therapeutic efficacy [28]. The most important explanation of the lack in vitro-in vivo correlation of combination therapy is that there are marked differences between in vitro and in vivo conditions due to serum protein binding that affects drug’s efficiency [29]. Consequently, an in vitro synergistic or antagonistic combination may be additive or indifferent in vivo if the administered dose levels result in drug’s concentrations lower or higher that those at which synergism or antagonism was observed in vitro [28]. Finally, the conclusion of the present in vitro studies is that the combinations of **2**-linezolid, **4**-levofloxacin, **5**-linezolid, **6**-gentamicin, **6**-linezolid, and **7**-levofloxacin are applicable to in vivo synergy studies, genotoxicity/mutagenicity tests, and pharmacokinetic/pharmacodynamics analyses to provide more information on their mechanism of action, safety profile, and potential clinical relevance. Such experiments are now in progress in our laboratory, and their results will soon be presented. 

### 2.4. Enzymatic Assay

**Figure 1:**
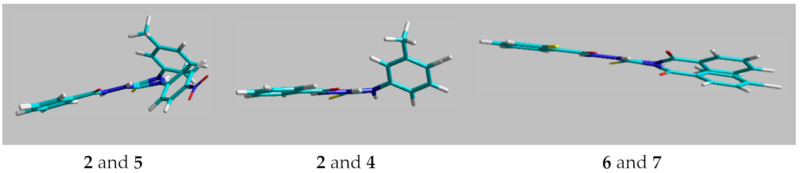
Overlay of structures of the 4-benzoylthiosemicarbazides **2**, **4**, **5**, and 4-arylthiosemicarbazides **6** and **7**. Note: The geometry of **1** is superimposable with **5**, whereas the geometry of **3** is superimposable with **2** and **4**. The geometries of **8** and **9** are superimposable with **6**.

Several studies have shown that closely related analogues and structurally similar scaffolds produce qualitatively the same biologic response and share a common function or mechanism of action [30]. Therefore, it is quite strange that our structurally and electronically closely related thiosemicarbazides tested in the present study exert synergistic effects with antibiotics that have totally different mechanisms. In essence, two 4-benzoylthiosemicarbazides with electron-donating *meta*-methyl substitution **2** and electron-withdrawing *para*-nitro substitution **5** share very similar molecular geometries, which only slightly differ in the orientation of the N4 aryl ring (Figure 1, left), and exerted synergistic effect when combined with linezolid that molecular mechanism involves inhibition of the initiation process of protein production [31]. In contrast, compound with *para*-chloro substitution **4**, which geometry is superimposable with **2** (Figure 1, middle), showed no synergy when combined with linezolid but acted synergistically with levofloxacin that involves inhibition of the enzymatic activity of DNA gyrase and topoisomerase IV; the enzymes essential for DNA replication, transcription, repair, and recombination. Likewise, within subclass of 4-arylthiosemicarbazides **6**–**9**, the chloro isomers **6** and **7** share very similar stretched geometries, only slightly different in the orientation of the N4 benzoyl ring (Figure 1, right), whereas produce quite different antibacterial response; isomer *para* **6** exerts synergistic effect in combinations with linezolid or gentamicin, while its isomer *meta* **7** acts synergistically in combination with levofloxacin.

Thus, based on SAR (structure activity relationship) analysis one can conclude that tested thiosemicarbazides **1**–**9** have a complex molecular-level antibacterial mechanism, which requires further investigation. When combined with our previous studies on molecular basis of antibacterial activity of thiosemicarbazides, these data provide further evidence that at least two molecular mechanisms for this class of compounds should be really expected. Thus far, the first one proposed by us assumed that antibacterial activity of thiosemicarbazides might be related to inhibition of bacterial topoisomerases [22,25,32,33]. Taking this fact into account, we have submitted **4** and **7** for enzyme inhibition assay against *S. aureus* bacterial topoisomerases (Figures S1–S3) to clarify the molecular mechanism involved in their synergistic effects observed in the combination with levofloxacin. No inhibitory actions, however, were recorded for both the compounds against DNA gyrase and topoisomerase IV. Finally, the inhibitory effects of **2**, **5**, and **6** that showed synergism in combination with linezolid or gentamycin against *S. aureus* DNA gyrase and topoisomerase IV were also assayed for comparison (Figures S1–S3). Surprisingly, the most potent synergist in our studies **6** at concentration of 100 µM was able to inhibit ~50% activity of *S. aureus* DNA gyrase (Table 4), suggesting that its anti-gyrase activity, although weak, may be a possible factor contributing to its synergism effect in combination with linezolid or gentamycin. For remaining thiosemicarbazides **1**, **3**, **8**, and **9** no inhibitory effects against *S. aureus* topoisomerase IV was observed (Figures S1–S3). Evidently, more research is needed to understand the molecular mechanism behind the identified synergy. Such studies are in progress in our laboratory.

## 3. Materials and Methods 

### 3.1. Chemistry

All commercial reactants and solvents were purchased from either Sigma-Aldrich (St. Louis, MO, USA) or Alfa Aesar (Karlsruge, Germany) with the highest purity and used without further purification. The melting points were determined on a Fischer-Johns block (Fischer Scientific, Schwerte, Germany) and are uncorrected. Elemental analyses were determined by a AMZ-CHX elemental analyzer (PG, Gdańsk, Poland) and are within ±0.4% of the theoretical values. ¹H-NMR spectra were recorded on a Bruker Avance (300 MHz) spectrometer (Bruker BioSpin GmbH, Rheinstetten, Germany). Analytical thin layer chromatography was performed with Merck 60F_254_ silica gel plates (Merck, Darmstadt, Germany) and visualized by UV irradiation (254 nm). Physicochemical characterizations of the compounds **1**–**6** were reported elsewhere [23,34]. The structures of compounds **7** and **8** are known (CAS numbers 443666-45-3 and 905231-62-1, respectively); however, there is no references reporting their preparation and physicochemical characterization; therefore, these data have been included into the manuscript.

### 3.2. General Procedure for Synthesis of the Thiosemicarbazides ***1***–***9***

A solution of 2,3-dichlorobenzoic hydrazide or 2-fluorobenzoic hydrazide (0.01 mol) and an equimolar amount of aryl or benzoyl isothiocyanate (0.01 mole) in anhydrous ethanol (25 mL) was heated under reflux for 10–30 min. After cooling, the solid formed was filtered off, dried, and crystallized from ethanol. 

*4-(3-chlorophenyl)-1-(2-fluorobenzoyl)thiosemicarbazide* (**7**). Yield: 84%. M.p. 158–160 °C. ^1^H-NMR (DMSO-*d*6) δ (ppm): 7.26–7.93 (m, 8H, 8 × CH_ar_); 9.91, 10.06, 10.41 (3s, 3H, 3 × NH). Anal. calc. for C_14_H_11_ClFN_3_OS (%): C 51.93, H 3.42, N 12.98. Found: C 51.79, H 3.71, N 12.89.

*1-(2-fluorobenzoyl)-4-(3-trifluoromethylphenyl)thiosemicarbazide* (**8**). Yield: 81%. M.p. 176–178 °C. ^1^H-NMR (DMSO-*d*6) δ (ppm): 7.37–7.91 (m, 8H, 8 × CH_ar_); 10.02, 10.03, 10.14 (3s, 3H, 3 × NH). Anal. calc. for C_15_H_11_F_4_N_3_OS (%): C 50.42, H 3.10, N 11.76. Found: C 50.35, H 3.32, N 11.88. Anal. (C, H, N).

*4-(3-bromophenyl)-1-(2-fluorobenzoyl)thiosemicarbazide* (**9**). Yield: 86%. M.p. 166–168 °C. ^1^H-NMR (DMSO-*d*6) δ (ppm): 7.30–7.91 (m, 8H, 8 × CH_ar_); 9.89, 10.04, 10.41 (3s, 3H, 3 × NH). Anal. calc. for C_14_H_11_BrFN_3_OS (%): C 45.67, H 3.01, N 11.41. Found: C 45.82, H 3.22, N 11.17. Anal. (C, H, N).

### 3.3. In Vitro Evaluation of Antibacterial Activity

*Minimal inhibitory concentration* (*MIC*) of each antibacterial agents was determined by standardized broth microdilution method with inoculum of 5 × 10^5^ CFU/mL and incubated for 24 h at 35 °C according to the recommendation of CLSI (Clinical Laboratory Standards Institute) [35]. The antibacterial activity of the thiosemicarbazides **1**–**9** and antibiotics (amoxicillin, gentamicin, levofloxacin, linezolid, and vancomycin) was tested on the Gram-positive strains (Microbiologics, St. Cloud, MN, USA) (*S. aureus* ATCC 25923, *S. aureus* ATCC 6538, *S. epidermidis* ATCC 12228, *B. subtilis* ATCC 6633, *B. cereus* ATCC 10876, *M. luteus* ATCC 10240), the Gram-negative strains (Microbiologics, St. Cloud, MN, USA) (*E. coli* ATCC 25922, *K. pneumoniae* ATCC 13883, *P. mirabilis* ATCC 12453, *P. aeruginosa* ATCC 9027), and clinical isolates of *S. aureus* (Medical University of Lublin, Lublin, Poland) (MSSA-1, MSSA-2, MSSA-3, MSSA-4, MRSA-1). Similarly to previous experiments with the thiosemicarbazides **1**–**5**, cefuroxime was used as positive control antibiotic [23], whereas sterile distilled water served as negative controls.

### 3.4. Fractional Inhibitory Concentration Indices (FICIs)

Interaction of the thiosemicarbazides **1**–**9** in combination with amoxicillin, gentamicin, levofloxacin, linezolid or vancomycin against *S. aureus* ATCC 25923 were analyzed using the checkerboard broth dilution method to determine the fractional inhibitory concentration indices (FICIs), calculated as: FIC = MIC of drug A in combination/MIC of drug A alone + MIC of drug B in combination/MIC of drug B alone. The calculated FICIs were interpreted as synergistic (FIC ≤ 0.5), additive (0.5 < FIC < 1), indifferent (1 ≤ FIC < 4.0), or antagonistic (FIC ≥ 4.0) [36,37]. The checkerboard assay included ciprofloxacin-carvedilol combination as positive control [38], whereas cefuroxime in combination with 4-(4-bromophenyl)-1-(3-fluorobezoyl)thiosemicarbazide served as negative control [39]. The *Z*-factor value for the bioassay was 0.683.

### 3.5. Cytotoxicity Assay

Cytotoxic properties of the compounds were evaluated using human foreskin fibroblast cells Hs27 (ATCC CRL-1634) (ATCC, Manassas, VA, USA) that were cultured in DMEM-high glucose (Sigma Aldrich, St. Louis, MO, USA). Medium was supplemented with 10% fetal bovine serum (FBS, Sigma Aldrich), 100 U/mL of penicillin, and 100 mg/mL of streptomycin (PenStrep, Sigma Aldrich, St. Louis, MO, USA). Cells were incubated at 37 °C in a humidified atmosphere of 5% CO_2_. Stock solutions of the tested compounds were prepared dissolving in a sterile dimethyl sulfoxide (DMSO) to the concentration of 50 mg/mL. The cells were seeded into 96-well sterile plates (Nunc) at a cell density of 1 × 10^5^ cells/mL. After 24 h of incubation, the medium was removed from each well, and then cells were incubated for the next 24 h with tested compounds (in a fixed dose of 25 µg/mL) in DMEM-high glucose containing 2% FBS. Control cells were cultured only with medium containing 2% addition of FBS. Cytotoxicity of the compounds was evaluated using MTT assay, which is based on the conversion of 3-(4,5-dimethylthiazol-2-yl)-2,5- diphenyltetrazolium bromide (MTT) into dark-blue formazan crystals. Briefly, after 24-h incubation of cells with varying concentrations of the tested compounds, culture medium was removed from the plates. Cells were washed with PBS, and then 100 µL of medium containing 10% MTT solution (5 mg/mL) was added to each well. Cells were incubated for the next 3 h at 37 °C in an atmosphere of 5% CO_2_. Next, 100 µL (per well) of 10% sodium dodecyl sulfate (SDS) buffer solution was added to dissolve formazan crystals and after an overnight incubation the absorbance was measured at 570 nm using a microplate reader (Epoch, BioTek Instruments, Inc., Winooski, VT, USA). *Z*-factor value for the bioassay was 0.996.

### 3.6. Inhibition of Bacterial type IIA Topoisomerases

*Supercoiling assay*: The assays were performed using *S. aureus* Gyrase Supercoiling Assay Kits (Inspiralis, Norwich, UK). Briefly, supercoiled pBR322 plasmid DNA (0.5 µg) was incubated with 1 unit of gyrase, in the dedicated supercoiling assay buffer supplied by the manufacturer, in the presence of varying concentrations of the test compounds. Reactions were carried out at 37 °C for 1 h and then terminated by the addition of equal volume of 2 × STOP Buffer (40% sucrose, 100 mM Tris-Cl pH 7.5, 1 mM EDTA, and 0.5 mg/mL bromophenol blue) and chloroform/*iso*-amyl alcohol. Samples were vortexed, centrifuged, and run through a 15 cm 1% agarose gel in TAE buffer (40 mM Tris-acetate, 2 mM EDTA) for 3 h at 50 V. Gels were stained with ethidium bromide and visualized under UV light.

*Inhibition of decatenation activity of S. aureus topoisomerase IV*: The assay was performed using *S. aureus* topoisomerase IV decatenation kits (Inspiralis, Norwich, UK). Interlinked kDNA substrate (0.5 µg) was incubated with 1 unit of topoisomerase IV (Inspiralis, Norwich, UK), in the dedicated decatenation assay buffer supplied by the manufacturer, in the presence of varying concentrations of the test compounds. Reactions were carried out at 37 °C for 1 h and then terminated by the addition of equal volume of 2 × STOP Buffer and chloroform/*iso*-amyl alcohol. Samples were vortexed, centrifuged, and run through a 15 cm 1% agarose gel in TAE buffer for 1.5 h at 80 V. Gels were stained with ethidium bromide and visualized under UV light. 

### 3.7. Computational Details

Conformational search was carried out using the molecular mechanics level with Amber99 force field [40], as implemented in HyperChem 8.0.3 (Hypercube, Gainesville, FL, USA) [41].

## 4. Conclusions

The checkerboard method was used to evaluate synergistic interactions between nine thiosemicarbazides **1**–**9** and five conventional antibiotics against *S. aureus* ATCC 25923. For these experiments, amoxicillin, gentamicin, levofloxacin, linezolid, and vancomycin were selected to represent the five antimicrobial classes most commonly used in clinical practice. With one exception of **7**-vancomycin combination, none of the thiosemicarbazide-antibiotic combinations tested had an antagonistic effect, showing promising results with respect to a combination therapy. The synergic effect was observed for the **2**-linezolid, **4**-levofloxacin, **5**-linezolid, **6**-gentamicin, **6**-linezolid, and **7**-levofloxacin combinations. No interactions were seen in combination of the thiosemicarbazide with gentamicin or vancomycin, whereas all combinations with linezolid acted in additive or synergism, except for **6**-gentamicin and **7**-linezolid. The thiosemicarbazide **6** showed a clear preference for the potency; it affected synergistically in combinations with gentamicin or linezolid and additively in combinations with amoxicillin, levofloxacin, or vancomycin. In further studies, the results of enzymatic assay confirmed that anti-gyrase activity of **6** may be a possible factor contributing to its synergism effect in combination with linezolid or gentamycin.

## Figures and Tables

**Table 1:** Antibacterial data (MIC, µg/mL) for 4-benzoylthiosemicarbazides **1**–**5**, 4-arylthiosemicarbazides **6**–**9**, and antibiotics.

	1	2	3	4	5	6	7	8	9	AMX	GN	LEV	LZD	VA
*S. a.* ATCC 6538	15.63	7.81	15.63	15.63	1.95	250	31.25	15.63	62.50	0.12	0.98	0.24	0.98	0.49
*S. a.* ATCC 25923	15.63	7.81	15.63	31.25	1.95	500	62.50	15.63	62.50	0.12	1.95	0.24	1.95	0.98
*S. e.* ATCC 12228	7.81	3.91	7.81	7.81	1.95	250	31.25	7.82	31.25	7.81	0.12	0.12	0.49	0.98
*B. s.* ATCC 6633	3.91	0.49	3.91	0.98	0.98	500	15.63	7.82	15.63	0.12	0.24	0.12	0.98	0.24
*B. c.* ATCC 10876	3.91	1.95	3.91	3.91	0.98	31.25	31.25	7.82	15.63	>250	15.63	0.12	0.98	0.98
*M. l.* ATCC 10240	1.95	0.98	0.98	1.95	0.49	7.82	7.82	7.82	7.82	0.24	0.98	0.98	0.98	0.24

Note: *S. a.—S. aureus*, *S. e.—S. epidermidis, B. s.—B. subtilis, B. c.—B. cereus, M. l.—M. luteus.* AMX*—*amoxicillin, GN*—*gentamicin, LEV—levofloxacin, LZD—linezolid, and VA—vancomycin. MICs for 4-benzoylthiosemicarbazides **1**–**5** against reference strains of Gram-positive bacteria are taken from Reference [23]. MICs for **1**–**9** and antibiotics against clinical isolates of *S. aureus* are presented in Table S1; all the thiosemicarbazides **1**–**9** were inactive against these clinical isolates (MICs ≥ 62.5 µg/mL). All the thiosemicarbazides **1**–**9** were inactive against Gram negative strains (*E. coli* ATCC 25922, *K. pneumoniae* ATCC 13883, *P. mirabilis* ATCC 12453, and *P. aeruginosa* ATCC 9027), as well.

**Table 2:** Viability of human foreskin fibroblast Hs27 cells (CRL-1634) determined by MTT assay after 24 h of treatment with 4-benzoylthiosemicarbazides (**1**–**5**) and 4-arylthiosemicarbazides (**6**–**9**).

	1	2	3	4	5	6	7	8	9	LEV
**IC_50_** **(µg/Ml)**	>25	>25	>25	>25	>25	>25	>25	>25	>25	>25
**Viability at 25 µg/mL (%)** *	78.48	76.68	80.97	80.72	70.91	96.84	93.21	65.24	63.11	100.51

Note: LEV—levofloxacin; * Mean from 3 independent measurements done in triplicate (SD values were in the range of ±5% of the reported values).

**Table 3:** In vitro evaluation of antibacterial combinations.

Compound	+ AMX	+ GN	+ LEV	+ LZD	+ VA
	FIC Effect	FIC Effect	FIC Effect	FIC Effect	FIC Effect
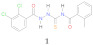	0.750 additive	1.125 indifferent	1.490 Indifferent	0.750 additive	1.500 indifferent
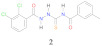	0.562 additive	1.062 indifferent	1.031 indifferent	0.281 synergistic	1.063 indifferent
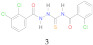	0.750 additive	0.750 additive	1.000 additive	0. 563 additive	1.250 indifferent
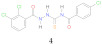	0.508 additive	1.500 indifferent	0.498 synergistic	0.563 additive	1.500 indifferent
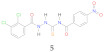	1.250 indifferent	2.125 indifferent	1.500 indifferent	0.375 synergistic	3.000 indifferent
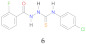	0.563 additive	0.375 synergistic	0. 625 additive	0.500 synergistic	0.625 additive
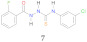	0.750 additive	1.125 indifferent	0.373 synergistic	1.031 indifferent	31.254 antagonistic
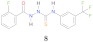	1.500 indifferent	1.266 indifferent	1.000 additive	0.750 additive	1.500 indifferent
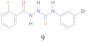	1.250 indifferent	1.125 indifferent	0.748 additive	0.750 additive	1.500 indifferent

Note: FIC is interpreted as synergistic (FIC ≤ 0.5), additive (0.5 > FIC ≤ 1.0), indifferent (1 < FIC ≤ 4), or antagonistic (FIC > 4.0). FIC—fractional inhibitory concentration indices, AMX—amoxicillin, GN—gentamicin, LEV—levofloxacin, LZD—linezolid, and VA—vancomycin.

**Table 4:** Inhibitory activity of **6** and **7** against *S. aureus* DNA gyrase.

Compound	*S. aureus* Gyrase DNA Inhibition (%) ± SD	
	50 µM	100 µM
**6**	30.76 ± 3.19	49.22 ± 3.18
**7**	23.84 ± 5.02	33.14 ± 1.04
**LEV**	n.d.	92.38 ± 3.74

Note: *S. aureus* gyrase DNA inhibition was quantified densitometrically from two independent experiments and expressed as mean ± SD. n.d.—not determined.

## Supplementary Materials

The following are available online, Figure S1: Inhibition of decatenation activity of *S. aureus* topoisomerase IV by compounds **1**–**5** and levofloxacin. Figure S2: Inhibition of decatenation activity of *S. aureus* topoisomerase IV by compounds **6**–**9** and levofloxacin. Figure S3: Inhibition of decatenation activity of *S. aureus* topoisomerase IV by compounds **1**–**9** and ciprofloxacin. Figure S4: The TLC (thin layer chromatography) chromatograms for 4-benzoylthiosemicarbazides (**1**–**5**) and 4-arylthiosemicarbazides (**6**–**9**). Table S1: Antibacterial data (MIC, µg/mL) for 4-benzoylthiosemicarbazides **1**–**5**, 4-arylthiosemicarbazides **6**–**9**, and antibiotics against clinical isolates of *S. aureus*.
