# Supplementary material for: Synergistic Effects of Thiosemicarbazides with Clinical Drugs against S. aureus
Source: Molecules. 2020 May 14;25(10):2302. doi: 10.3390/molecules25102302 (PMC7288171; doi:10.3390/molecules25102302)
Supplement: Supplementary file 1 [file molecules-25-02302-s001.pdf]

Synergistic effects of thiosemicarbazides with clinical drugs against *S. aureus*

Beata Chudzik-Rząd [1], Anna Malm [1], Nazar Trotsko [2], Monika Wujec [2], Tomasz Plech [3], and Agata Paneth [2,*]

[1] Department of Pharmaceutical Microbiology with Laboratory for Microbiological Diagnostics, Faculty of Pharmacy, Medical University of Lublin, Chodźki 1, 20-093 Lublin, Poland; BeChudz@poczta.fm anna.malm@umlub.pl

[2] Department of Organic Chemistry, Faculty of Pharmacy, Medical University of Lublin, Chodzki 4a, 20-093 Lublin, Poland; monika.wujec@umlub.pl agata.paneth@umlub.pl nazar.trotsko@umlub.pl

[3] Department of Pharmacology, Faculty of Health Sciences, Medical University of Lublin, Chodźki 4a, 20-093 Lublin, Poland; tomasz.plech@umlub.pl

* Correspondence: agata.paneth@umlub.pl (A.P.)

Supplementary Material

Figure S1. Inhibition of decatenation activity of *S. aureus* topoisomerase IV by compounds **1**-**5**.
Figure S2. Inhibition of decatenation activity of *S. aureus* topoisomerase IV by compounds **6**-**9** and levofloxacin.
Figure S3. Inhibition of decatenation activity of *S. aureus* topoisomerase IV by compounds **1**-**9** and ciprofloxacin.
Figure S4. The TLC chromatograms for 4-benzoylthiosemicarbazides (**1**-**5**) and 4-arylthiosemicarbazides (**6**-**9**).
Table S1. Antibacterial data [MIC, μg/mL] for 4-benzoylthiosemicarbazides **1**-**5**, 4-arylthiosemicarbazides **6**-**9**, and antibiotics against clinical isolates of *S. aureus*.

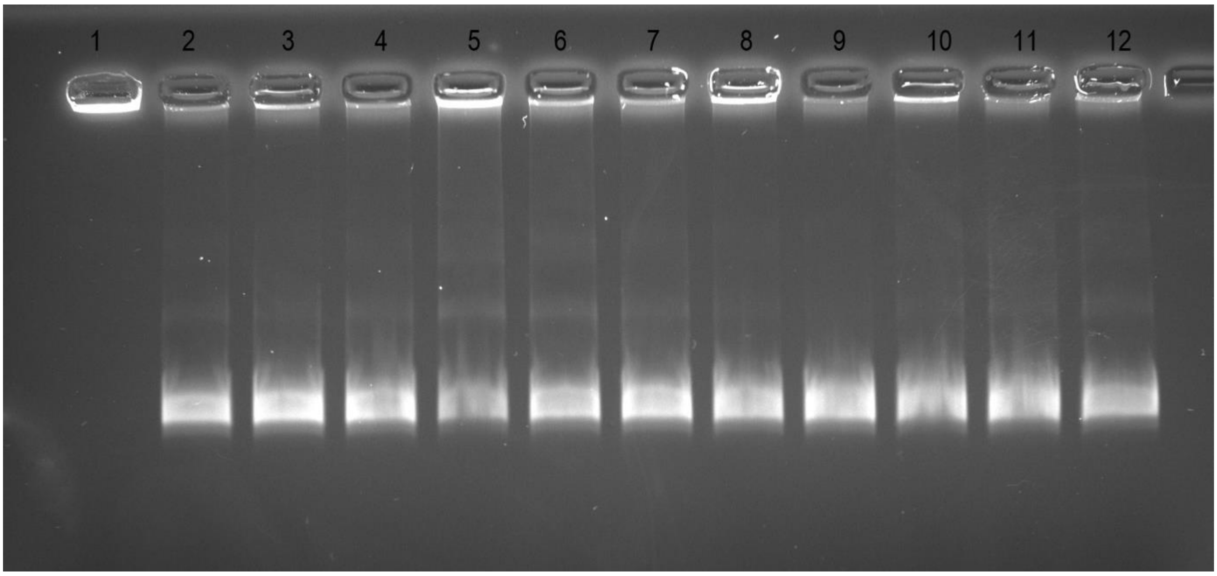

Figure S1. Inhibition of decatenation activity of *S. aureus* topoisomerase IV by compounds **1**-**5**.
Description of the wells:
1 – substrate (kDNA) without enzyme – negative control (catenated DNA)
2 – substrate (kDNA) with enzyme – positive control (decatenated DNA)
3 – compound **1** (50 μM)
4 – compound **1** (100 μM)
5 – compound **2** (50 μM)
6 – compound **2** (100 μM)
7 – compound **3** (50 μM)
8 – compound **3** (100 μM)
9 – compound **4** (50 μM)
10 – compound **4** (100 μM)
11 – compound **5** (50 μM)
12 – compound **5** (100 μM)

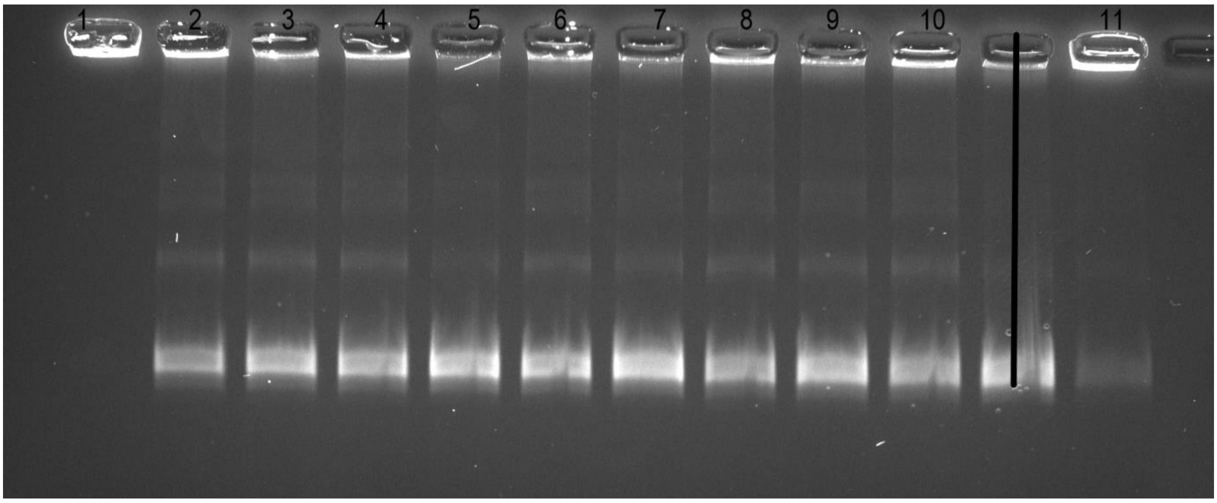

Figure S2. Inhibition of decatenation activity of *S. aureus* topoisomerase IV by compounds **6**-**9** and levofloxacin.
Description of the wells:
1 – substrate (kDNA) without enzyme – negative control (catenated DNA)
2 – substrate (kDNA) with enzyme – positive control (decatenated DNA)
3 – compound **6** (50 μM)
4 – compound **6** (100 μM)
5 – compound **7** (50 μM)
6 – compound **7** (100 μM)
7 – compound **8** (50 μM)
8 – compound **8** (100 μM)
9 – compound **9** (50 μM)
10 – compound **9** (100 μM)
11 – **levofloxacin** (50 μM)

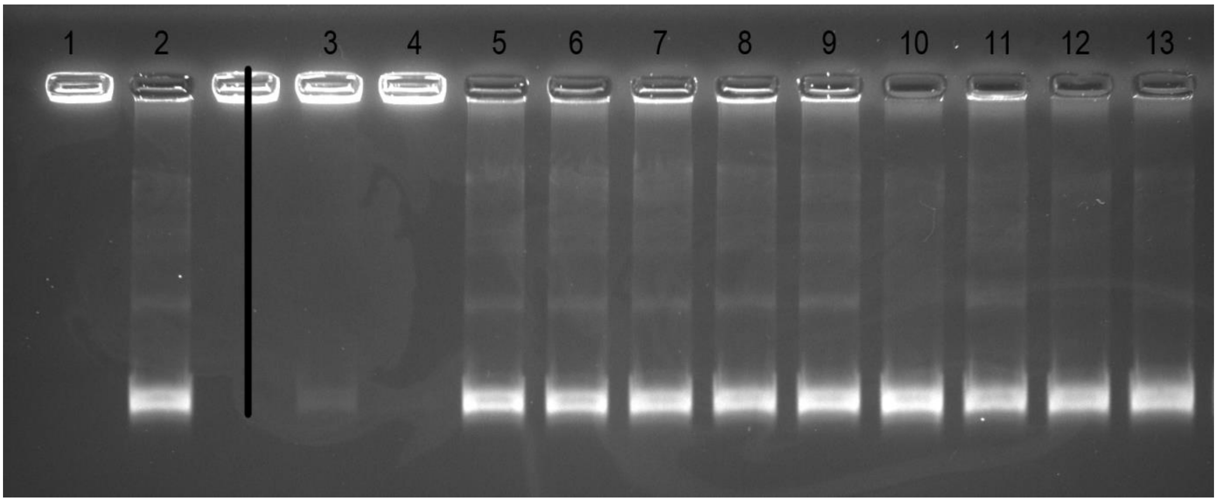

Figure S3. Inhibition of decatenation activity of *S. aureus* topoisomerase IV by compounds **1**-**9** and ciprofloxacin. Description of the wells:

1 – substrate (kDNA) without enzyme – negative control (catenated DNA)
2 – substrate (kDNA) with enzyme – positive control (decatenated DNA)
3 – **ciprofloxacin** (50 μM)
4 – **ciprofloxacin** (100 μM)
5 – compound **1** (100 μM)
6 – compound **2** (100 μM)
7 – compound **3** (100 μM)
8 – compound **4** (100 μM)
9 – compound **5** (100 μM)
10 – compound **6** (100 μM)
11 – compound **7** (100 μM)
12 – compound **8** (100 μM)
13 – compound **9** (100 μM)

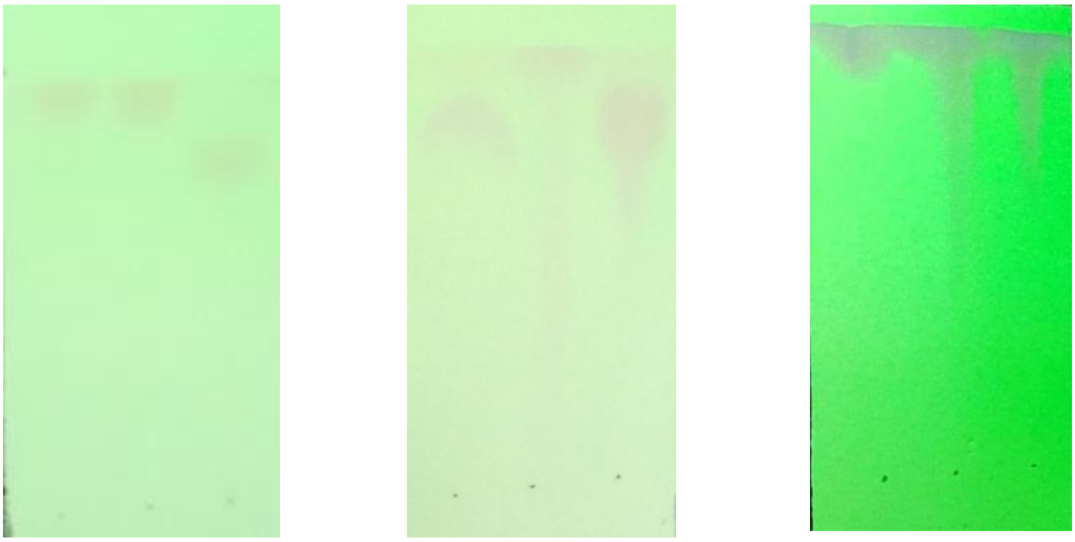

A B C

Figure S4. The TLC chromatograms for 4-benzoylthiosemicarbazides (**1**-**5**) and 4-arylthiosemicarbazides (**6**-**9**). Note: the chromatograms were developed in chloroform-ethanol (10:1) and visualised under UV light of 254 nm. **A**: the thiosemicarbazides **1**-**3** (*from left to right*); **B**: the thiosemicarbazides **4**-**6** (*from left to right*); **C**: the thiosemicarbazides **7**-**9** (*from left to right*).

Table S1. Antibacterial data [MIC, µg/mL] for 4-benzoylthiosemicarbazides **1-5**, 4-arylthiosemicarbazides **6-9**, and antibiotics against clinical isolates of *S. aureus*.

	**1**	**2**	**3**	**4**	**5**	**6**	**7**	**8**	**9**	**AMX**	**GN**	**LEV**	**LZD**	**VA**
**MSSA-1**	>500	>500	>500	>500	>500	250	125	62.5	62.5	1.95	0.98	0.12	1.95	0.49
**MSSA-2**	>500	>500	>500	>500	>500	>500	>500	>500	>500	0.12	0.49	0.24	1.95	0.49
**MSSA-3**	>500	>500	>500	>500	>500	250	125	>500	>500	0.98	7.81	0.12	0.98	0.49
**MSSA-4**	>500	500	250	500	500	125	125	>500	125	31.25	1.95	0.12	1.95	0.49
**MRSA-1**	125	500	125	>500	250	500	500	>500	125	31.25	0.98	31.25	1.95	0.98

Note: **AMX** – amoxicillin, **GN** – gentamicin, **LEV** – levofloxacin, **LZD** – linezolid, **VA** – vancomycin.
